# Miscibility and Nanoparticle Diffusion in Ionic Nanocomposites

**DOI:** 10.3390/polym10091010

**Published:** 2018-09-10

**Authors:** Argyrios Karatrantos, Yao Koutsawa, Philippe Dubois, Nigel Clarke, Martin Kröger

**Affiliations:** 1 Materials Research and Technology, Luxembourg Institute of Science and Technology, 5, Avenue des Hauts-Fourneaux, L-4362 Esch-sur-Alzette, Luxembourg; yao.koutsawa@list.lu; 2 Center of Innovation and Research in Materials and Polymers (CIRMAP), Laboratory of Polymeric and Composite Materials, University of Mons & Materia Nova Research Centre, Place du Parc 20, B-7000 Mons, Belgium; philippe.dubois@umons.ac.be; 3 Department of Physics and Astronomy, University of Sheffield, Sheffield S3 7RH, UK; n.clarke@sheffield.ac.uk; 4 Polymer Physics, Department of Materials, ETH Zurich, Leopold-Ruzicka-Weg 4, CH-8093 Zurich, Switzerland

**Keywords:** ionic nanocomposites, miscibility, chain dimensions, nanoparticle diffusion

## Abstract

We investigate the effect of various spherical nanoparticles in a polymer matrix on dispersion, chain dimensions and entanglements for ionic nanocomposites at dilute and high nanoparticle loading by means of molecular dynamics simulations. The nanoparticle dispersion can be achieved in oligomer matrices due to the presence of electrostatic interactions. We show that the overall configuration of ionic oligomer chains, as characterized by their radii of gyration, can be perturbed at dilute nanoparticle loading by the presence of charged nanoparticles. In addition, the nanoparticle’s diffusivity is reduced due to the electrostatic interactions, in comparison to conventional nanocomposites where the electrostatic interaction is absent. The charged nanoparticles are found to move by a hopping mechanism.

## 1. Introduction

In the past three decades, polymer nanocomposites [1,2,3,4], where spherical, cylindrical or plate-like nanoparticles are finely distributed and dispersed [5,6,7] in a polymer matrix, have become of growing importance to industry and academia due to their advanced mechanical [8,9,10], thermal [11,12,13], tribological [14], rheological [15], and electrical properties [16] comparable to polymer blends. Because nanoparticles are increasingly being added to polymers, there is a motivation to explore how nanoparticles impact polymer dynamics [17,18,19,20], structure [21,22,23,24], morphology [25,26,27,28,29], stability [30], and ultimately how these features are correlated and how they impact macroscopic properties [1]. Nanoparticle dispersion [31,32,33,34,35,36] (high degree of distribution/dispersion is needed for effective reinforcement [28,33] in the matrix) allows property “tuning” [24] and provides appropriate functionalities. There are three main ideas on how to achieve better nanoparticle dispersion. The first is to tether the chains on the surface of the nanoparticle, of linear size [7,37,38] or larger than the quarter length of polymer matrix chains [39,40]. The second is to achieve a chemical favorable interaction between nanoparticle and polymer matrix [41,42,43,44]. The third idea is to let the interaction between nanoparticles and chains to be of ionic nature [5,6]. Nanoparticle aggregation has been observed for conventional polymer nanocomposites with weak interactions, such as polystyrene–silica nanocomposite [45], for nanocomposites with an oligomeric matrix [41,46], possibly for the repulsive nanoparticles composite of poly(ethylene-propylene) (PEP)–silica (in which the transmission electron microscopy (TEM) data, were not reported [47]), and also for the polyurethane–silica nanocomposite when the electrostatic interactions are absent [48,49]. Nowadays, dispersion of high concentrations of surface-functionalised spherical nanoparticles (up to 50 vol% [47,50,51,52,53,54]) is accessible.

While there is a substantial amount of research on nanocomposites containing bare or grafted nanoparticles [55,56,57,58], exhibiting tethered chains on the surface of the nanoparticle, there is only a limited amount of research in ionic polymer nanocomposites. In these materials, the nanoparticles are ionically functionalized and react with a polymer with a functionality of the opposite charge [59] (either end-terminated or grafted along the chain). Oppositely charged ions interact with energies greater than the thermal energy. The presence of oppositely charged ions at the polymer/nanofiller interphase can promote dispersion according to Refs. [48,49]. This is a new class of ionic polyurethane nanocomposites that combines sulfonate-modified nanosilicas reinforcement with the reversibility of ionic interactions. Such nanocomposite materials have presented an increase of the storage modulus and toughness [48,49].

Nanoparticle mobility [60,61] is a feature that has been observed in nanocomposites [62,63,64,65]. This mobility and the interphase region [66] can be altered by changing the nanoparticle size [66,67] and polymer–nanoparticle interactions [66,68]. The nanoparticle (colloid) diffusivity in a polymer matrix can be predicted by the Stokes–Einstein relation [69] and depends on the viscosity of the matrix, η, and the nanoparticle radius, *R*, according to $D_{\mathrm{SE}}=k_{B}T/6\pi \eta R$, in agreement with experiments [70] and the theoretical scaling by Brochard-Wyart and de Gennes [71], when the nanoparticle radius, *R*, is greater than the tube diameter [72], $d_{T}$ (R≥ 3.5–5$\;d_{T}$) [64]. The Stokes–Einstein relation is however invalid when nanoparticles are smaller (R≤ 2$\;d_{T}$) as shown recently by simulations [61,73,74] and experiments [64,67]. Such small nanoparticles are very mobile and alter [61,75] polymer diffusion [62,76,77,78].

To the best of our knowledge, fundamental research on ionic polymer nanocomposites to understand and investigate the ionic interactions, nanoparticle mobility on the interphasial region, and nanoparticle dispersion state of the nanocomposite have not been performed so far. Exceptions are the coarse grained model by Hong et al. [79,80] for nanoparticle ionic liquids, where nanoparticles diffuse like in a polymer solution [81,82,83] while chains diffuse faster than nanoparticles, as well as the studies in ionomer nanocomposites [59,84], polymer charged solutions [85,86] and polymer gels [87,88].

We set out to investigate how the ionic (electrostatic) interaction between nanoparticles and polymers impact the nanoparticle dispersion state, polymer structure/dimensions and nanoparticle mobility (diffusion). The rest of this paper is organized as follows. In Section 2, the methodology and simulation details of the present study are described. Subsequently, in Section 3.1, the structure of nanoparticles in the polymer melt is investigated and compared to that of experiments [48]. In Section 3.2, the polymer dimensions of unentangled and weakly entangled chains is calculated. In Section 3.3, the diffusion of nanoparticles of different diameter, in the various molecular weight polymer matrices, is calculated subject to dilute nanoparticle loading, and compared to the Stokes–Einstein formula and experiment. Finally, in Section 4, conclusions are presented.

## 2. Methodology

**Figure 1:**
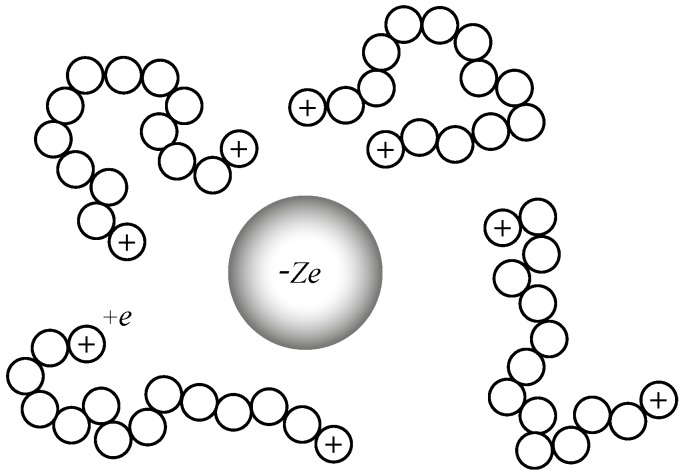
Schematic of ionic nanocomposite: Chain ends are positively charged (+e) and nanoparticles are negatively charged (−Ze).

To address these fundamental questions, we use the molecular dynamics method [89,90,91,92] of a coarse grained semiflexible polymer model (Kremer–Grest) [93,94] with nanoparticles, using the GROMACS 4.6.7 version (University of Groningen, Groningen, Holland). A schematic of the ionic nanocomposite of polymers with charged chain ends and ionic nanoparticles is shown in Figure 1.

The classical Newton–Langevin equations that govern the motion of the particles is [89,93]:

(1) $$m_{i}\frac{dv_{i}}{dt}=-\nabla V_{i}-\Gamma \frac{dr_{i}}{dt}+W_{i}\left(t\right),$$

where $V_{i}$ is the potential experienced by particle *i*, and $m_{i}$ its mass; Γ is the friction coefficient and $W_{i}$ describes the random force which essentially is a Gaussian white noise with zero mean acting on each particle. The total deterministic force $f_{i}$ on particle *i* is the gradient of the potential $V_{i}$ given by a sum of four terms:

(2) $$V_{i}=\sum_{j\neq i}\left(V_{ij}^{\mathrm{LJ}}+V_{ij}^{\mathrm{Coulomb}}+V_{ij}^{S}+V_{ij}^{B}\right).$$

The truncated, purely repulsive Lennard–Jones (LJ) potential $V_{ij}^{\mathrm{LJ}}$, whose corresponding force acts along the line between the centres of mass of two particles [95], is given by

(3) $$V_{ij}^{\mathrm{LJ}}=4\epsilon_{ij}\left(\frac{\sigma_{ij}^{12}}{{r_{ij}}^{12}}-\frac{\sigma_{ij}^{6}}{{r_{ij}}^{6}}\right),\;r_{ij}\leq r_{c}=2^{1/6}\sigma_{ij},$$

where $r_{ij}$ represents the distance between particles *i* and *j*, $\epsilon_{ij}$ is the characteristic interaction energy between particle *i* and particle *j*. The combination rules $\epsilon_{ij}={\left(\epsilon_{i}\epsilon_{j}\right)}^{1/2}$ and $\sigma_{ij}=\left(\sigma_{i}+\sigma_{j}\right)/2$ [95] are used; for monomers: $\epsilon_{m}=\sigma_{m}=m_{m}=1$, for nanoparticles: $\epsilon_{n}=1$, $\sigma_{n}=2R$, $R/\sigma_{m}=$ 2 or 4, $m_{n}=0.85\times 4\pi R^{3}/3$. For monomer (m) – nanoparticle (p) interactions, a more repulsive LJ potential is selected $V_{ij}^{\mathrm{LJ}}=4\epsilon_{\mathrm{mn}}{\left(\sigma_{\mathrm{mn}}/r_{ij}\right)}^{12}-2\epsilon_{\mathrm{mn}}{\left(\sigma_{\mathrm{mn}}/r_{ij}\right)}^{6}$ for $r_{ij}\leq r_{c}$. In addition, the coulombic interaction is given by

(4) $$V_{ij}^{\mathrm{Coulomb}}=\frac{q_{i}q_{j}}{4\pi \epsilon_{r}\epsilon_{0}r_{ij}}$$

with $q_{m}=+e$ and $q_{n}=-Ze$. The particle mesh Ewald (PME) method [96,97,98] has been used to treat the long range electrostatics with a Fourier-spacing of 0.12 nm and an order of interpolation 4. The dielectric constant of the coulombic part is $\epsilon_{r}=50$ [99,100]. The Bjerrum length $l_{B}/\sigma_{m}=Zq_{i}q_{j}/\epsilon_{r}k_{B}T<1$ (for R=2) signals weak electrostatic strength between monomers and nanoparticles. Adjacent monomers within polymer chains are connected using the finitely extendable nonlinear elastic (FENE) spring potential [93]

(5) $$V_{ij}^{S}=-\frac{1}{2}k{R_{0}}^{2}\ln\left(1-\frac{r_{ij}^{2}}{{R_{0}}^{2}}\right),$$

where, in applying Equation (5), the sum in Equation (2) is over all particles *j* to which particle *i* is permanently connected. The maximum bond length and spring coefficient were set to $R_{0}=1.5$ and k=30, respectively, as in previous works on neutral polymers [93]. The stiffness of the polymer chains is controlled by a cosine harmonic bending potential [101], which acts on consecutive bonds along the chain,

(6) $$V_{ijk}^{B}=\frac{1}{2}k_{\theta }{\left(\cos\theta_{ijk}-\cos\theta_{0}\right)}^{2},$$

where $\theta_{ijk}$ is the bending angle between three consecutive beads. We use the equilibrium value $\theta_{0}=109.5^{\circ }$, and the bending constant $k_{\theta }=25$ [101]. By increasing the intramolecular stiffness [72] of the polymer chain, the entanglement length [94,102] is decreased to a value of $N_{e}\approx 48$ (as predicted by the modified S-coil estimator) similar to Ref. [73] ($N_{e}\approx 45$).

The simulations of the polymer nanocomposites consisted of spherical nanoparticles in a dense polymer melt. They were performed in a simulation cell starting from relaxed configurations of conventional non-ionic nanocomposites [61], using the isothermal isobaric (NPT) ensemble. The pressure calculated for the N= 200 polymer melt was $P^{*}=P\sigma_{m}^{3}/\epsilon_{m}=$ 4.864 [68]. That pressure was used to perform all the nanocomposite systems simulations in the NPT ensemble. The linear size of the simulation cell was always larger than the root mean square end-to-end distance of the polymer chains. To set the temperature at $T^{*}=k_{B}T/\epsilon =1$ and pressure at $P^{*}=4.864$, the Langevin thermostat with a friction constant $\Gamma =0.5{\tau_{\mathrm{LJ}}}^{-1}$ and the Berendsen barostat were used with time constant $2\tau_{\mathrm{LJ}}$, respectively. The equations of motion were integrated using the Leap frog algorithm [103] with a time step equal to $0.005\tau_{\mathrm{LJ}}$ for polymer melts, and a time step of $0.002\tau_{\mathrm{LJ}}$ for nanocomposite simulations with R=2 ($0.001\tau_{\mathrm{LJ}}$ for nanocomposite simulations with R=4), where $\tau_{\mathrm{LJ}}={\left(m\sigma_{m}{}^{2}/k_{B}T\right)}^{1/2}$ is the LJ time unit. The duration of the simulation production runs were between $4\times 10^{4}-4\times 10^{7}\tau_{\mathrm{LJ}}$ depending on the length of molecules. Details of the ionic nanocomposite systems studied (nanoparticle volume fraction: ϕ, number of nanoparticles: $N_{n}$, nanoparticle charge: −Ze, nanoparticle radius: *R*, Bjerrum length $l_{B}$) are summarized in Table 1.

## 3. Results and Discussion

### 3.1. Nanoparticle and Polymer Structure

**Figure 2:**
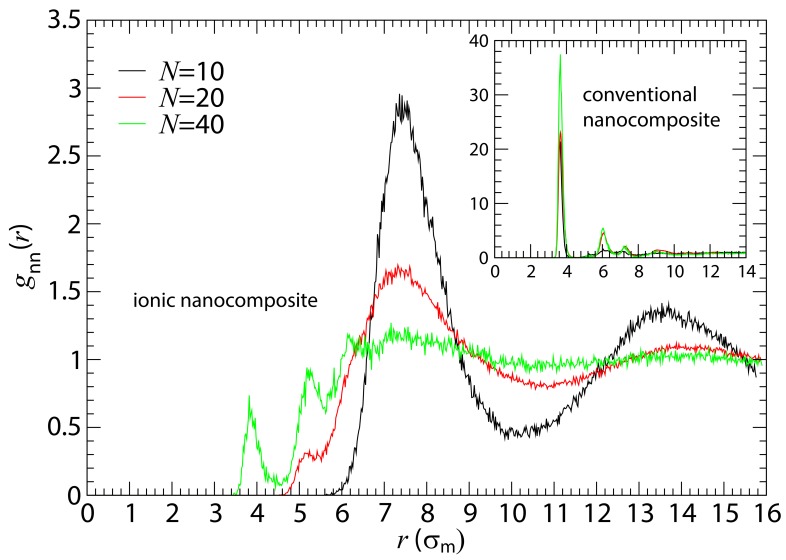
Nanoparticle–nanoparticle radial distribution functions (RDF) $g_{\mathrm{nn}}\left(r\right)$ in the ionic nanocomposites for different polymer matrices with nanoparticles (R=2) at ≈ 10% volume fraction. Inset: Nanoparticle-nanoparticle radial distribution functions in conventional nanocomposites with nanoparticles (R=2) for different polymer matrices at 10% volume fraction. The RDF for the ionic nanocomposite with N=200 shows a similar behavior with those in the conventional nanocomposites (results shown in Figure S3).

We first focus on the analysis of local nanoparticle and polymer structure [66] in these ionic nanocomposites. In Figure 2, we show the nanoparticle–nanoparticle radial distribution function $g_{\mathrm{nn}}\left(r\right)$, for different polymerization degrees of the chains, in ionic nanocomposites with small nanoparticles (R=2). On one hand, for conventional nanocomposites in the presence of repulsive monomer–nanoparticle interactions, there is a very high probability for the nanoparticles to be in contact with each other rather than with monomers (inset of Figure 2), which implies nanoparticle aggregation. On the other hand, attractive (electrostatic) monomer–nanoparticle interaction helps the nanoparticles to be well dispersed in the polymer matrix. For low nanoparticle loading (ϕ=10%), there are no nanoparticle contacts if the number of monomers in the chain is N=10 or N=20, while, if *N* is increased further, some nanoparticle–nanoparticle contacts emerge, as can be seen from the first peak of $g_{\mathrm{nn}}\left(r\right)$ in Figure 2. For a longer polymer matrix (N=200), nanoparticle dispersion is not achieved for our polymers who carry charges at their terminals only (results shown in the supplementary information, Figure S3).

For slightly higher nanoparticle loading (ϕ=17.7%), a similar behavior appears ($g_{\mathrm{nn}}\left(r\right)$ data shown in Figure S1), whereas for even higher loading at ϕ≈24% (Figure S2), nanoparticle aggregation occurs. Experiments [48] reported ϕ=20% as an upper limit for which nanoparticles could still be dispersed effectively; beyond that threshold, nanoparticle aggregation occured. Upon increasing the nanoparticle radius to R=4, poor dispersion is also observed in conventional nanocomposites, c.f. Figure S3. Thus, in nanocomposites containing nanoparticles of radius to R=2 or R=4, the nanoparticles form aggregated clusters in the absence of electrostatic interaction. However, when the monomer–nanoparticle electrostatic attraction is present, there are not any (or few) nanoparticle contacts for loading below 20%, for the oligomers matrices with N=10 and N=20, as can be seen from the simulations ($g_{\mathrm{nn}}\left(r\right)$ in Figure 2) and TEM pictures [48]. In the simulations, the nanoparticles are well dispersed in the oligomeric matrix (the same behavior is observed for nanoparticles of radius R=4) for nanoparticle loading below 20% in agreement with the experimental observations [48,49]. Nanoparticle dispersion is a result of the incorporation of charges in the nanocomposite and not due to changes in $R/R_{g}$. It is worth noting that, at loading below 20%, nanoparticle dispersion occurs at the Bjerrum length $l_{B}\geq 0.24\;\sigma_{m}$. In conventional (non-ionic) nanocomposites, a phase separation has been observed when $R_{g}\leq R$ in agreement with experiments [41,46].

**Figure 3:**
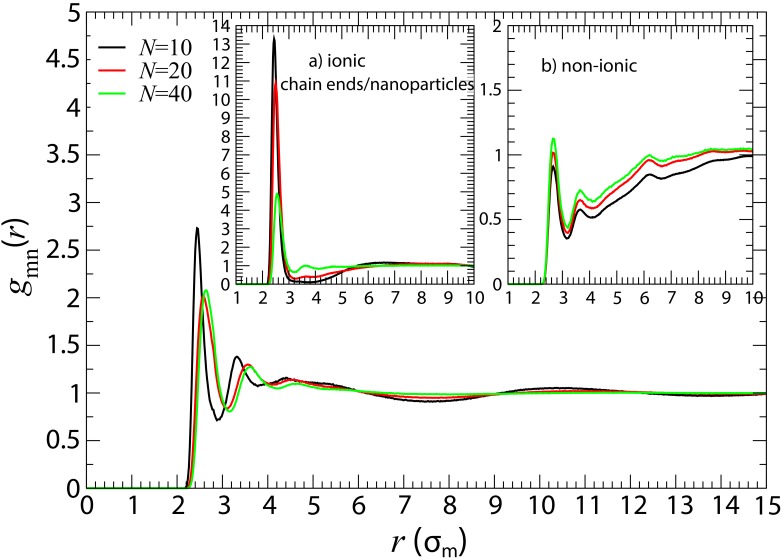
Monomer-nanoparticle radial distribution functions (RDF) $g_{\mathrm{mn}}\left(r\right)$ in ionic nanocomposites for different polymer matrices with nanoparticles (R=2) at ≈ 10% volume fraction. Inset: (**a**) chain end monomer–nanoparticle radial distribution functions for the very same systems of the outset; (**b**) monomer–nanoparticle radial distribution functions in conventional (non-ionic) nanocomposites with nanoparticles (R=2) for different polymer matrices at 10% volume fraction. The RDF for the ionic nanocomposite with N=200 shows a similar behavior with those in the conventional nanocomposites (results shown in Figure S3).

In Figure 3, we show the monomer–nanoparticle radial distribution function $g_{\mathrm{mn}}\left(r\right)$, for different polymerization degrees, in ionic nanocomposites with small nanoparticles (R=2). As can be seen from Figure 3, $g_{\mathrm{mn}}\left(r\right)$ exhibits a layering structure. The high monomer density of the layers establishes a well defined interphase [104] between nanoparticles and polymer melt whose structure differs from that of the amorphous polymer melt. By dispersing charged nanoparticles in the polymer matrix the polymer density around the nanoparticles increases, but the interphase layer is thin, as can be seen by the enhanced first peak of $g_{\mathrm{mn}}\left(r\right)$, compared to $g_{\mathrm{mn}}\left(r\right)$ in conventional nanocomposites with repulsive nanoparticles (inset b in Figure 3). Moreover, the chain end monomers/nanoparticle contacts are enhanced in oligomer matrices due to the electrostatic attraction that leads to the dispersion of charged nanoparticles (inset a in Figure 3).

### 3.2. Polymer Dimensions

We now turn attention to the polymer dimensions [54] analysis of ionic nanocomposites. The radius of gyration $R_{g}$ of a molecule, defined as the average squared distance between monomers in a given conformation and the molecule’s center of mass is given by [68,105]

(7) $$\langle R_{g}^{2}\left(N\right)\rangle =\frac{1}{N}\langle \sum_{i=1}^{N}{\left(r_{i}-r_{\mathrm{cm}}\right)}^{2}\rangle ,$$

where $r_{\mathrm{cm}}=N^{-1}\sum_{i=1}r_{i}$ is the center of mass of the chain. The radii of gyration of the polymer melt simulated systems are given in Table 2.

We begin by focusing on polymer dimensions of nanocomposites with nanoparticles (R= 2) dispersed in polymer matrices with varying polymerization degrees (N= 10–200). The nanoparticles are phase separated in the conventional nanocomposites with repulsive nanoparticles (of R=2,4), and there is no change on radius of gyration values. In particular, for the case of conventional nanocomposites containing short polymers (N= 10, 20, 40) as matrix, polymers remain unaltered compared with their bulk values, at low nanoparticle loading (ϕ= 10%). We do not find any evidence for polymer contraction at any nanoparticle loading. For entangled PEP polymers filled with silica nanoparticles (which is a repulsive system), a polymer contraction of 12% above percolation (ϕ= 50%) was reported [47].

**Figure 4:**
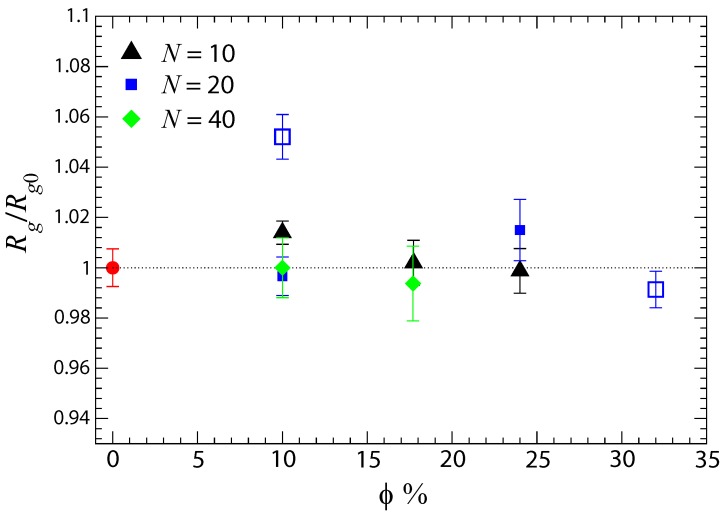
Radius of gyration $R_{g}$ of end-charged polymers in the presence of oppositely charged nanoparticles, relative to their $R_{g0}$, nanoparticle-free melt (red filled circle at ϕ=0%) value. Results for the ionic nanocomposite as a function of nanoparticle volume fraction for a polymer matrix with polymerization degree (i) N= 10 (triangles up); and (ii) N= 20 (blue squares), N= 40 (green diamonds), N= 200 (triangles down). Filled symbols are for R=2 whereas open symbols are for R=4. Error bars are estimated by the standard deviation values of $R_{g}$ and propagated for the ratio $R_{g}/R_{g0}$. The error bar for the melt corresponds to N=20 matrix.

In ionic nanocomposites containing charged nanoparticles of radius R=2, oligomers (N=10) are expand their conformation only at low nanoparticle loading (ϕ=10%), as long as $R_{g}<R$. Polymer expansion does not appear in ionic nanocomposites at higher nanoparticle loading as can be seen in Figure 4 and Table 2. Additionally, we have observed that the average radius of gyration of short polymers (N=10 and N=20) increases, in comparison to its bulk value, in ionic nanocomposites but only at low nanoparticle loading (10%) and as long as $R_{g}<R$ (Figure 4). However, $R_{g}$ doesn’t change in comparison to its bulk value for higher nanoparticle loadings or if $R_{g}\geq R$. We can thus conclude that short polymers in ionic nanocomposites with $R_{g}<R$, are perturbed at low nanoparticle loading such as ϕ=10% but remain unperturbed at higher nanoparticle loading.

In addition, we have calculated the primitive paths of the polymer chains with charges located at their chain ends, and upon ignoring the nanoparticles (phantom limit) as in [106], using the Z1 algorithm [107]. The chains’ entanglement lengths $N_{e}$ as predicted by different estimators, for conventional and ionic nanocomposites, are given in Table 3. The polymer entanglement length does not change (outside the error margin) with the addition of the nanoparticles.

### 3.3. Nanoparticle Diffusion

While in other theoretical studies [61,73,109,110] only neutral nanoparticle diffusion [74,111] has been explored, we investigate here, apparently for the first time, diffusion of charged nanoparticles in polymer matrices. The nanoparticle diffusivities of the simulated nanocomposite systems are calculated from the mean square displacement measurements (Figure S5) as follows:

(8) $$D_{0}=\frac{1}{6}\lim_{t\to \infty }\frac{d}{dt}\langle |r_{i}\left(t\right)-r_{i}{\left(0\right)|}^{2}\rangle ,$$

where $\langle |r_{i}\left(t\right)-r_{i}\left(0\right){|}^{2}\rangle$ is the time dependent mean square displacement of nanoparticle *i*, averaged over the ensemble of nanoparticles, and $r_{i}\left(t\right)$ denotes the center position of a nanoparticle *i* at time *t*.

**Figure 5:**
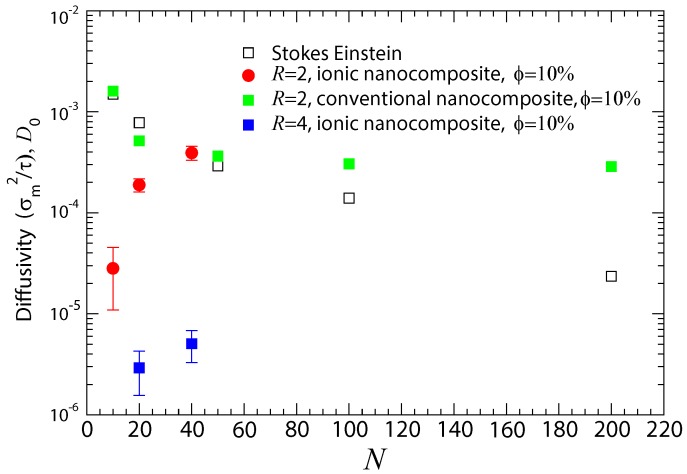
Dependence of nanoparticle (R=2 and R=4) diffusivity $D_{0}$ according to Equation (8) in unentangled and entangled polymer matrices at 10% volume fraction: (i) Stokes–Einstein relation predictions (open symbols); (ii) conventional nanocomposite (green squares) [61]; (iii) ionic nanocomposites, R=2 (red circles); and (iv) ionic nanocomposites, R=4 (blue squares). Missing error bars on the diffusion coefficients are smaller than the symbol sizes.

The nanoparticle diffusivity in different polymer matrices can be inferred from Figure 5. The diffusion of such small sized nanoparticles in conventional nanocomposites [67,110,112] reaches a plateau in entangled polymer matrices for N>100 [61,73,81,111], in agreement with the Generalized Langevin Equation (GLE) theory [113,114,115] and theoretical predictions by de Gennes [116]. He argued that bulk viscosity does not capture the behavior of surrounding flows near nanoparticles, and thus the nanoparticle diffusion is decoupled from the Stokes–Einstein relation.

It can be seen, from the data, that such nanoparticles, smaller than the tube diameter $d_{T}$ ($d_{T}\approx$ 10.3$\;\sigma_{m}$ for our polymer model), deviate more from the Stokes–Einstein predictions with increasing the molecular weight of the polymer matrix. Clearly, it can be seen that, in the dilute nanoparticle regime (ϕ≈ 10%), small charged nanoparticles (R= 2) diffuse slower in unentangled matrices than the Stokes–Einstein relation predicts and than those in conventional nanocomposites in unentangled matrices, where the dispersion has been achieved [61] (in that study also the attractive term of the Equation (3) was included between nanoparticles and polymer, $r_{c}=2.4\;\sigma_{\mathrm{mn}}$). This is due to the electrostatic attraction between the polymer chains and nanoparticles. The Stokes–Einstein relation can be valid for the nanoparticles diffusivity in conventional nanocomposites containing very short and unentangled polymers at the dilute regime, in agreement with experiments [65]. Since the nanoparticle diameter is smaller than the entanglement strand, such dispersed nanoparticles can diffuse inside the mesh of polymers, faster in comparison to the Stokes–Einstein predictions, due to local viscosity [61,74,75,117], but this is not the case in ionic nanocomposites. In these materials, the electrostatic strength as controlled by the Bjerrum length affects the diffusion of the nanoparticles.

**Figure 6:**
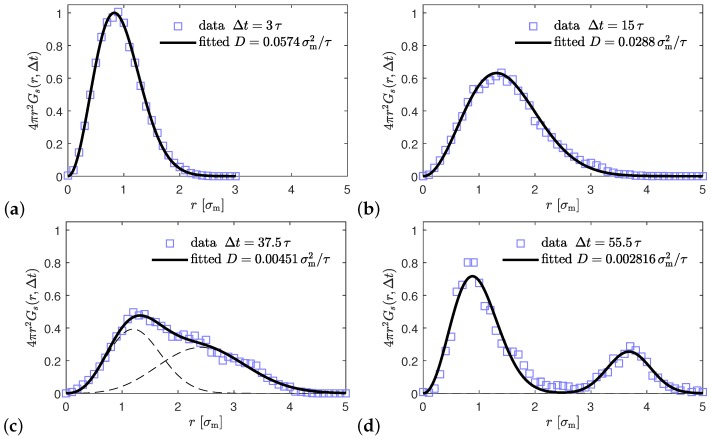
Van Hove function $G_{s}\left(r,\Delta t\right)$ for nanoparticles in nanocomposite with ϕ=10%, nanoparticle radius R=4, nanoparticle charge Z=75, polymer matrix N=40, at four different Δt (in units of $\tau =1000\tau_{\mathrm{LJ}}$). Blue symbols are data from the simulations, and solid lines are fitting of Equation (10) in the two upper panels (**a**,**b**) for Δt≤15τ, while the two bottom panels (**c**,**d**) for larger time intervals Δt≥37.5τ can only be fitted by a superposition of two Gaussians. The individual contributions are shown by black dashed lines (basically invisible for Δt=55.5τ). The mentioned values for *D* correspond to the first peak in (**c**,**d**).

Furthermore, we calculated the displacement distribution of the nanoparticles at different times. Such distribution can be obtained from the self-part of the van Hove function (VHF),

(9) $$G_{s}\left(r,\Delta t\right)={\langle \delta \left[r-|r_{i}\left(t_{0}+\Delta t\right)-r_{i}\left(t_{0}\right)|\right]\rangle }_{t_{0}},$$

which gives the probability density to find a nanoparticle at a distance *r* from the initial position after a time interval Δt. The probability is normalized as $\int G_{s}\left(r,\Delta t\right)d^{3}r=1$. As can be seen from Figure 6, for nanocomposites with nanoparticles R= 4 and matrix N=40 at small time intervals 3τ and 15τ ($\tau =1000\tau_{\mathrm{LJ}}$), the data can be fitted to a a Gaussian function (10) with a peak value corresponding to the most probable traveled distance during that time interval Δt

(10) $$G_{s}\left(r,\Delta t\right)={\left(4\pi D\Delta t\right)}^{-3/2}e^{-r^{2}/4D\Delta t}.$$

For larger time intervals Δt=37.5τ and 55.5τ, the displacement distribution of the nanoparticles can only be fitted using a bimodal superposition of Gaussians, which can be attributed to a hopping motion [118] of the nanoparticles. It means that charged nanoparticles effectively hop in space. In particular, the area of the secondary local peak in the displacement distribution can become larger at a long time interval Δt, indicating that the hopping is performed more easily on longer time scales (diffusion regime). This phenomenon is also observed in fullerene ($C_{60}$)/polystyrene or polypropylene nanocomposites as was shown recently by atomistic simulations [112]. Similar behavior appears for the other ionic nanocomposites studied, where dispersion has achieved (results shown in Figures S6–S9).

## 4. Conclusions

To summarize, we investigated the structure and conformations of polymers and nanoparticle diffusion, for the first time in ionic nanocomposites containing small spherical nanoparticles up to high volume fraction, using a coarse grained model for nanoparticles and polymers by means of molecular dynamics simulations. We find that in nanocomposites with repulsive monomer–nanoparticle interaction, such as in conventional nanocomposites, the polymers and nanoparticles phase separate in agreement to experiments. However, in ionic nanocomposites and for short polymer chains matrix (N= 10, 20, 40), nanoparticle dispersion is achieved. This result has also been observed in qualitative agreement with the experimental data.

Additionally, we have observed that the average radius of gyration of ionic oligomer chains (N= 10, 20) with charged chain ends can be perturbed (expanded) by charged nanoparticles in ionic nanocomposites at low nanoparticle loading such as ϕ=10%, but only if $R_{g}<R$. However, these short polymer chains as well as their entangled counterparts do not alter their size compared to their melt values at the higher volume fractions studied, for both repulsive and charged nanoparticles. The nanoparticles are still mobile but their diffusion, which follows a hopping motion, is decreased due to the electrostatic strength between monomers and nanoparticles.

## Figures and Tables

**Table 1:** Nanoparticle volume fraction ϕ (%), nanoparticle radius *R*, number of spherical nanoparticles $N_{n}$, individual nanoparticle charge −Ze, polymerization degree *N* and number of such chains $N_{\mathrm{ch}}$, Bjerrum length $l_{B}/\sigma_{m}$, for ionic nanocomposite systems studied. Nanoparticle dispersion is detected for systems marked by a checkmark. The monomer radius is $\sigma_{m}/2=0.5$ in each case.

ϕ	*R*	$N_{n}$	*Z*	*N*	$N_{\mathrm{ch}}$	$l_{B}/\sigma_{m}$	Dispersion
10.0%	2	100	48	10	2400	0.96	✓
10.0%	2	100	24	20	1200	0.48	✓
10.0%	2	100	12	40	600	0.24	✓
10.0%	2	100	2.4	200	120	0.048	—
10.0%	4	8	150	20	600	3	✓
10.0%	4	8	75	40	300	1.5	✓
17.7%	2	100	24	10	1200	0.48	✓
17.7%	2	100	12	20	600	0.24	✓
17.7%	2	100	6	40	300	0.12	—
24.0%	2	300	8	20	1200	0.16	✓
24.0%	2	300	4	40	600	0.08	—
32.0%	4	50	48	20	1200	0.96	✓
32.0%	4	50	24	40	600	0.48	—

**Table 2:** Average radius of gyration (of $\sigma_{m}$ units) for both ionic and conventional polymer nanocomposite systems studied in the present simulations. Volume fraction ϕ. Nanoparticle radius *R*. Polymerization degree *N*. The nanocomposites systems in which nanoparticle dispersion has not been achieved have not been analyzed.

	System	ϕ	*R*	N=10	20	40
$R_{g0}$	(melt)	0%	–	1.574	2.363	3.466
$R_{g}$	(conventional)	10.0%	2	1.570	2.353	–
$R_{g}$	(charged)	10.0%	2	1.596	2.355	3.454
$R_{g}$	(charged)	10.0%	4	–	2.486	–
$R_{g}$	(charged)	17.7%	2	1.578	2.353	3.445
$R_{g}$	(charged)	24.0%	2	1.572	2.393	–
$R_{g}$	(charged)	32.0%	4	–	2.342	–

**Table 3:** Volume fraction ϕ, nanoparticle radius *R*, chain length *N*, end to end distance $R_{\mathrm{ee}}$, contour length of primitive path $L_{\mathrm{pp}}$, coil- and kink-based, classical and modified $N_{e}$ estimators [94,108], $a_{\mathrm{pp}}$ is the tube diameter of the ionic and conventional nanocomposites, $Z_{\mathrm{kinks}}$ is the number of interior kinks of the primitive path. All lengths, *R*, $R_{\mathrm{ee}}$, $L_{\mathrm{pp}}$, and $a_{\mathrm{pp}}$ are given in terms of $\sigma_{m}$ units. The other nanocomposites at higher nanoparticle loading with polymer matrix N=40 have not been analyzed this way, since nanoparticle dispersion has not been achieved.

						Coil-Based			Kink-Based		
System	ϕ	R	N	$R_{\mathrm{ee}}$	$L_{\mathrm{pp}}$	$a_{\mathrm{pp}}$	$N_{e}^{\mathrm{class}-S}$	$N_{e}^{\mathrm{mod}-S}$	$Z_{\mathrm{kinks}}$	$N_{e}^{\mathrm{class}-S}$	$N_{e}^{\mathrm{mod}-S}$
melt	0%	–	40	8.5 ± 0.1	9.9 ± 0.1	7.3	28.8 ± 0.6	74.8 ± 4.5	1.07 ± 0.04	19.1 ± 0.4	37.6 ± 1.5
charged	10.0%	2	40	8.7 ± 0.2	10.1 ± 0.2	7.4	28.8 ± 0.6	75.3 ± 4.4	1.03 ± 0.04	19.5 ± 0.4	39.0 ± 1.5
charged	10.0%	4	40	8.9 ± 0.2	10.5 ± 0.2	7.6	28.1 ± 0.6	72.0 ± 4.8	1.12 ± 0.05	18.6 ± 0.4	35.7 ± 1.6

## Supplementary Materials

The following are available online at http://www.mdpi.com/2073-4360/10/9/1010/s1, Figures S1–S5: RDF for ionic nanocomposites at different nanoparticle volume fractions and for various *N*, Figure S5: Mean square displacement for different ionic nanocomposites at ϕ=10%, Figures S6–S9: van Hove functions for ionic nanocomposites at ϕ=10%.
