# Supplementary material for: Miscibility and Nanoparticle Diffusion in Ionic Nanocomposites
Source: Polymers (Basel). 2018 Sep 10;10(9):1010. doi: 10.3390/polym10091010 (PMC6403637; doi:10.3390/polym10091010)
Supplement: Supplementary file 1 [file polymers-10-01010-s001.pdf]

Supplementary Materials: Miscibility and nanoparticle diffusion in ionic nanocomposites

Argyrios Karatrantos [1]*, Yao Koutsawa [1] Philippe Dubois [2], Nigel Clarke [3], and Martin Kröger, [4],

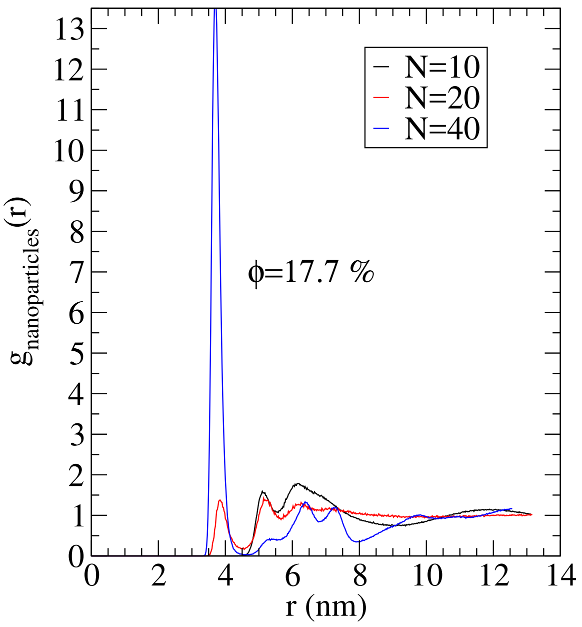

Figure S1. Nanoparticle-nanoparticle radial distribution functions (RDF) in the ionic nanocomposites for different polymer matrices ($N = 10, 20$) with nanoparticles ($R = 2$) at $\phi \approx 17.7\%$ volume fraction.

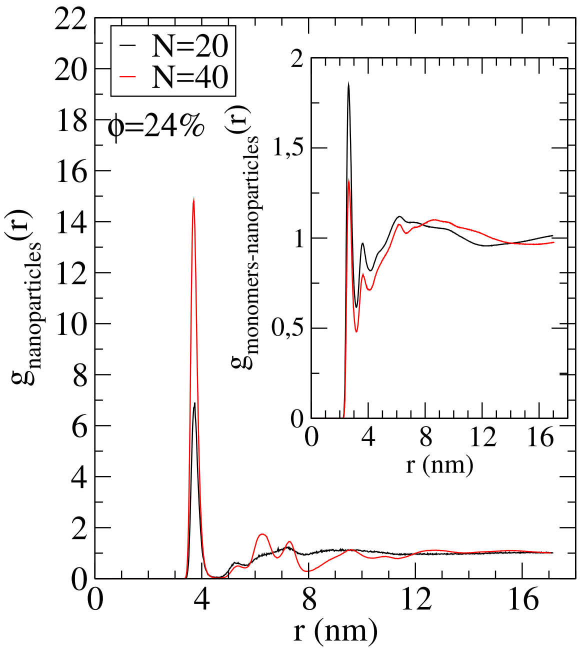

Figure S2. Nanoparticle-nanoparticle radial distribution functions (RDF) in the ionic nanocomposites for different polymer matrices ($N = 20, 40$) with nanoparticles ($R = 2$) at $\phi \approx 24\%$ volume fraction. Monomer-nanoparticle radial distribution function for the same system.

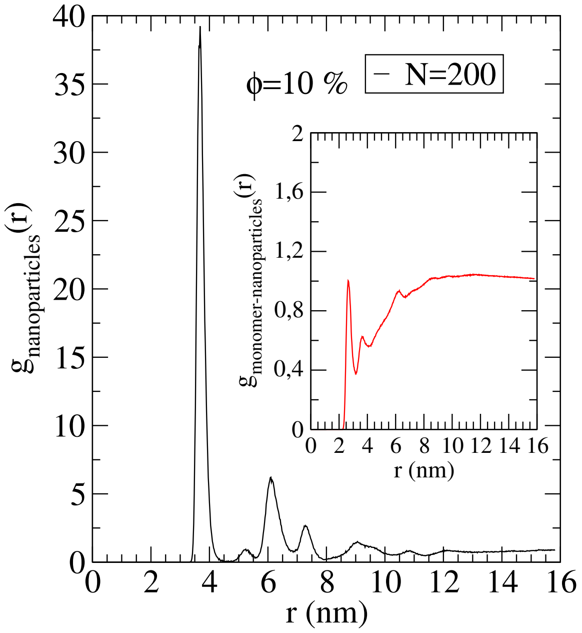

Figure S3. Nanoparticle-nanoparticle radial distribution functions (RDF) in the ionic nanocomposite for polymer matrix ($N = 200$) with nanoparticles ($R = 2$) at $\phi \approx 10\%$ volume fraction.Inset:Nanoparticle-monomer radial distribution function for the same system.

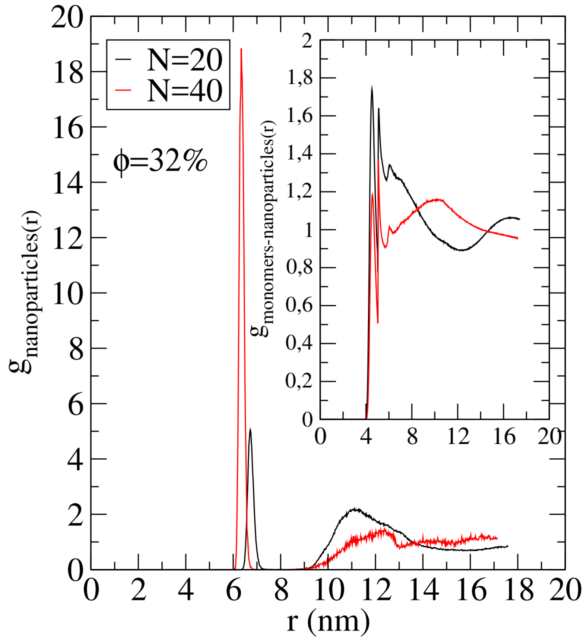

Figure S4. Nanoparticle-nanoparticle radial distribution functions (RDF) in the ionic nanocomposite for oligomeric polymer matrices ($N = 20, 40$) with nanoparticles ($R = 4$) at $\phi \approx 32\%$ volume fraction. Monomer-nanoparticle radial distribution function for the same system

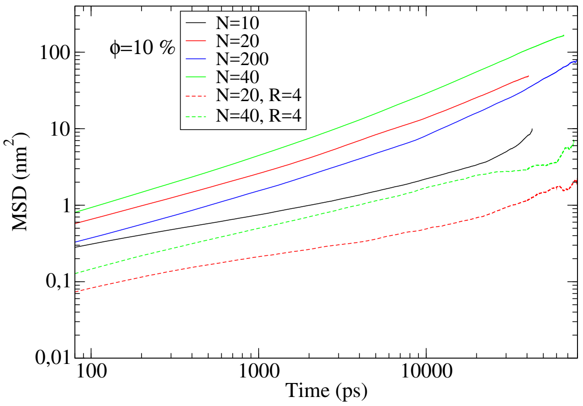

Figure S5. Nanoparticle ($R = 2$) mean square displacement in ionic nanocomposites for different polymer matrices at loading $\phi \approx 10\%$. Solid lines for nanoparticles $R = 2$. Dashed lines for nanoparticles $R = 4$.

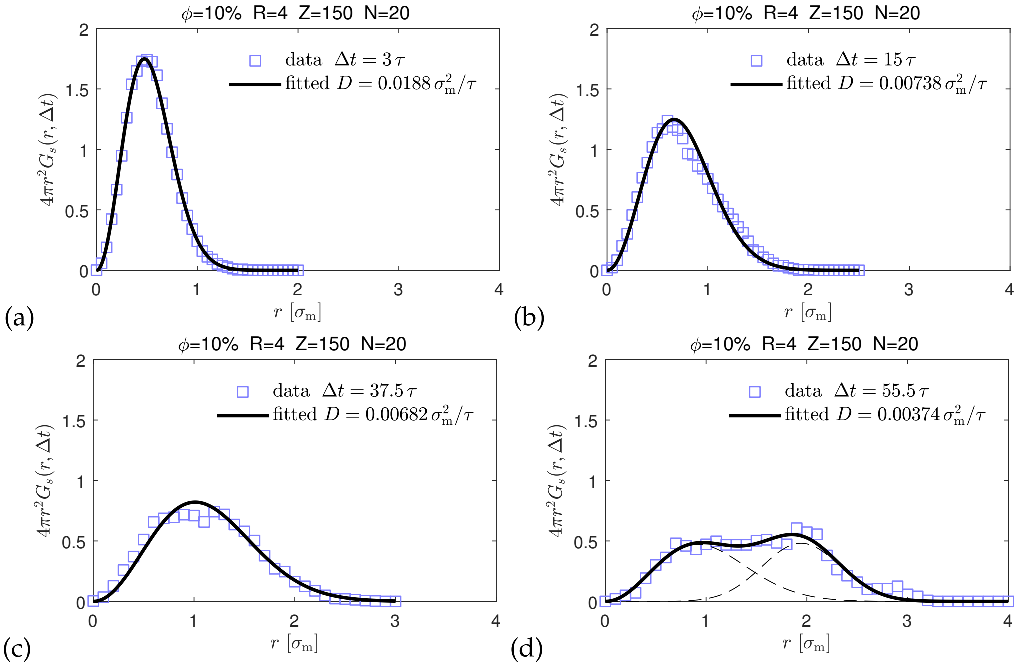

Figure S6. van Hove function $G_s(r, \Delta t)$ for nanoparticles in nanocomposite with $\phi = 10\%$, nanoparticle radius $R = 4$, nanoparticle charge $Z = 150$, polymer matrix $N = 20$, at four different Δt. Blue symbols are data from the simulations, and solid lines are fitting of Equation 10 in the first three panels (a)+(b)+(c) for $\Delta t \leq 37.5\tau$, while the right bottom panel (d) for larger time interval $\Delta t = 55.5\tau$ can only be fitted by a superposition of two Gaussians. The individual contributions are shown by black dashed lines. The mentioned values for D correspond to the first peak in (d).

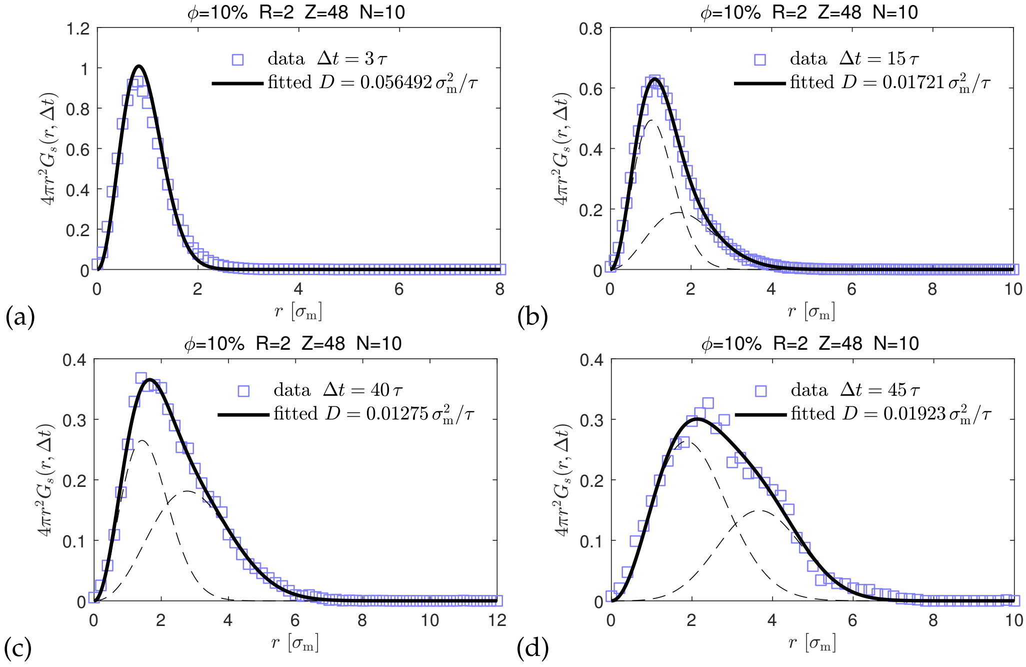

Figure S7. van Hove function $G_s(r, \Delta t)$ for nanoparticles in nanocomposite with $\phi = 10\%$, nanoparticle radius $R = 2$, nanoparticle charge $Z = 48$, polymer matrix $N = 10$, at four different Δt. Blue symbols are data from the simulations, and solid lines are fitting of Equation 10 in the two upper panels (a)+(b) for $\Delta t \leq 15\tau$, while the two bottom panels (c)+(d) for larger time intervals $\Delta t \geq 40\tau$ can only be fitted by a superposition of two Gaussians. The individual contributions are shown by black dashed lines. The mentioned values for D correspond to the first peak in (c)+(d).

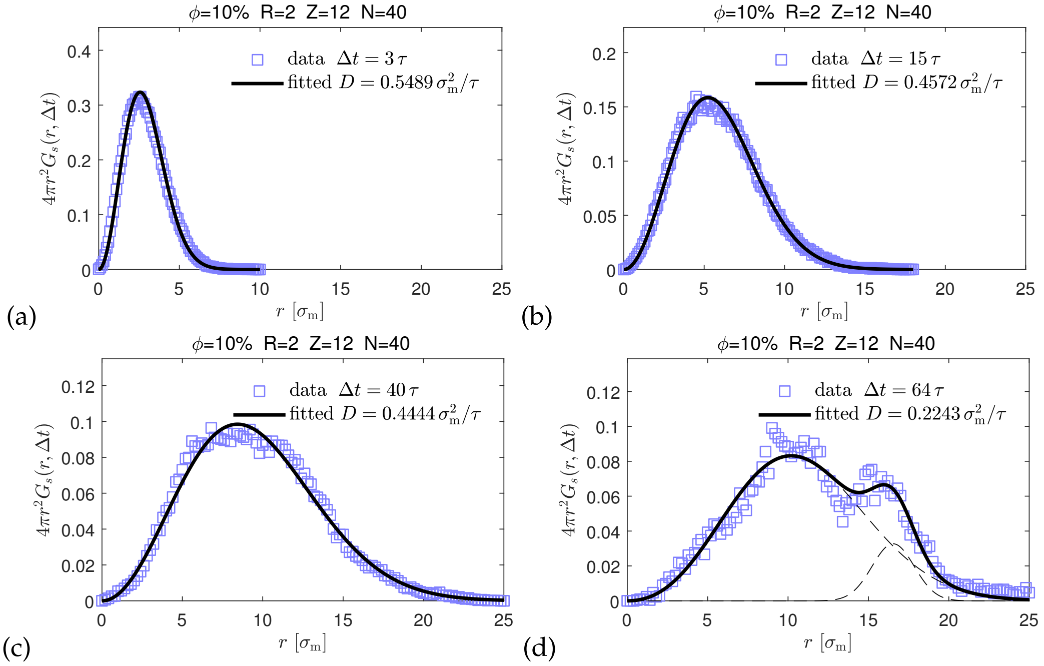

Figure S8. van Hove function $G_s(r, \Delta t)$ for nanoparticles in nanocomposite with $\phi = 10\%$, nanoparticle radius $R = 2$, nanoparticle charge $Z = 12$, polymer matrix $N = 40$, at four different Δt. Blue symbols are data from the simulations, and solid lines are fitting of Equation 10 in the first three panels (a)+(b)+(c) for $\Delta t \leq 40\tau$, while the right bottom panel (d) for larger time interval $\Delta t = 64\tau$ can only be fitted by a superposition of two Gaussians. The individual contributions are shown by black dashed lines. The mentioned values for D correspond to the first peak in (d).

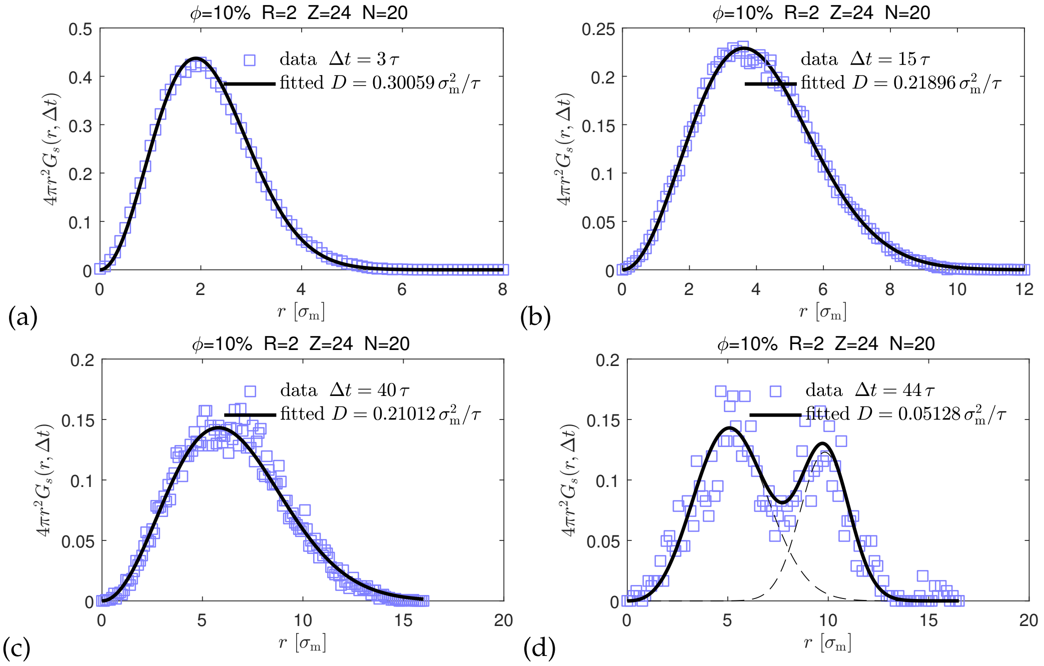

Figure S9. van Hove function $G_s(r, \Delta t)$ for nanoparticles in nanocomposite with $\phi = 10\%$, nanoparticle radius $R = 2$, nanoparticle charge $Z = 24$, polymer matrix $N = 20$, at four different Δt. Blue symbols are data from the simulations, and solid lines are fitting of Equation 10 in the first three panels (a)+(b)+(c) for $\Delta t \leq 40\tau$, while the right bottom panel (d) for larger time interval $\Delta t = 44\tau$ can only be fitted by a superposition of two Gaussians. The individual contributions are shown by black dashed lines. The mentioned values for D correspond to the first peak in (d).
